# Genetic Variation in *CYP2B6*, *UGT1A4* and Sulfotransferases Is Associated with Disease-Free Survival in South African Breast Cancer Patients Treated with Tamoxifen

**DOI:** 10.3390/jpm16040188

**Published:** 2026-03-31

**Authors:** Bianca Kruger, Emile R. Chimusa, Aron B. Abera, Jesmika Singh, Delva Shamley, Collet Dandara

**Affiliations:** 1 Platform for Pharmacogenomics Research and Translation (PREMED), South African Medical Research Council, Cape Town 7700, South Africa; krgbia001@myuct.ac.za (B.K.); sngjes003@myuct.ac.za (J.S.); 2 Pharmacogenomics and Drug Metabolism Research Group, Division of Human Genetics, Department of Pathology, Institute of Infectious Diseases and Molecular Medicine, Faculty of Health Sciences, University of Cape Town, Cape Town 7700, South Africa; 3 Bioinformatic and Multi-Omics Data Science Group, Faculty of Science and Environment, Northumbria University, Newcastle NE1 8ST, UK; emile.chimusa@northumbria.ac.uk; 4 Inqaba Biotechnical Industries, Pretoria 0002, South Africa; aron.abera@inqababiotec.co.za; 5 Division of Clinical Anatomy and Biological Anthropology, Department of Human Biology, Faculty of Health Sciences, University of Cape Town, Cape Town 7700, South Africa; delva.shamley@uct.ac.za

**Keywords:** Africa, breast cancer, *CYP2D6*, disease-free survival, pharmacogenetics, tamoxifen

## Abstract

**Background**: Tamoxifen is widely used in the treatment of hormone receptor-positive breast cancer and has been shown to successfully reduce recurrence and mortality rates. Nonetheless, variability in patient response to tamoxifen treatment is observed with up to 40% of patients experiencing recurrence. Genetic polymorphisms in pharmacogenes encoding enzymes involved in tamoxifen metabolism have been linked to some of this observed interindividual variability. The pharmacogenetics of tamoxifen in populations of African descent remain understudied, creating difficulties in pinpointing the primary factors behind the observed variable response. To address this gap, this study aimed to investigate the role of genetic variation in tamoxifen treatment outcomes in a South African cohort. **Methods**: Participants included 166 Mixed and African Ancestry breast cancer patients who had received tamoxifen treatment. Genetic characterization was performed for 53 single nucleotide polymorphisms (SNPs) and two copy number variations across eight drug-metabolizing enzymes, including cytochrome P450s (*CYP2D6*, *CYP3A4*, *CYP3A5*, *CYP2B6*), UDP-glucuronosyltransferases (*UGT1A4*), and sulfotransferases (*SULT1A1*, *SULT1E1*, *SULT2A1*). The association between genotypes and disease-free survival (DFS) was evaluated using Cox proportional hazards regression models. **Results**: The CYP2B6*1/*6 or *4/*9 genotype showed a nominal association with improved DFS (*p* = 0.049), with a similar trend observed for *UGT1A4* rs11888492. In contrast, *SULT1E1* rs3775779 heterozygosity showed a nominal association with reduced DFS (*p* = 0.044). *SULT1A1* SNPs (rs4149393, rs4149394, rs1042157) demonstrated trends toward reduced DFS. **Conclusions**: These exploratory findings highlight the need for more inclusive pharmacogenomic research and point to potential biomarkers for optimizing tamoxifen therapy in African populations.

## 1. Introduction

Breast cancer is the most frequently diagnosed cancer among women, representing nearly 30% of all new cancer diagnoses in Africa [1]. Africa exhibits the world’s highest breast cancer mortality rates [1]. In South Africa, breast cancer represents 25% of new cancers and 15% of cancer-related deaths among women [1]. These statistics underscore the need for improved breast cancer treatment strategies specifically tailored to African populations.

Tamoxifen is the standard endocrine therapy for hormone-receptor positive breast cancer, including tumors that are estrogen receptor-positive (ER+) and/or progesterone-receptor positive. ER+ disease constitutes around 55% of all breast cancer cases in Africa and over two-thirds of cases in South Africa [2,3]. Tamoxifen use varies widely across Africa, from 38% in Uganda to a substantial 93% in Cameroon [4]. In South Africa, tamoxifen is used in approximately 48% of breast cancer patients [4]. Notably, tamoxifen remains the only hormonal therapy approved by the United States Food and Drug Administration for treating ER+ breast cancer in premenopausal women. This is particularly relevant in African settings, where patients often present at a younger age and with advanced disease [5].

**Figure 1:**
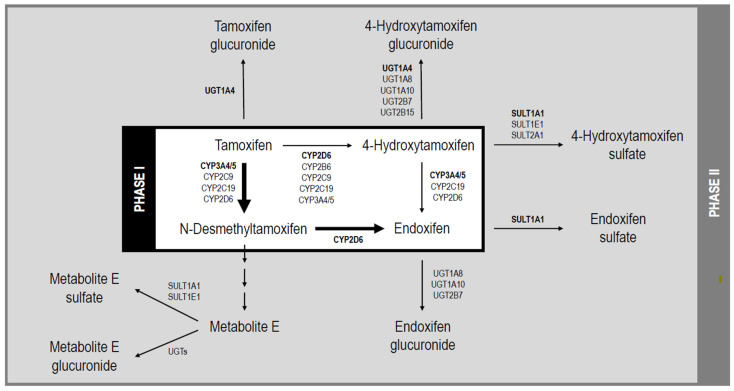
Metabolic pathway of tamoxifen. Major pathways of phase I metabolism via cytochrome P450 enzymes (in black box) are indicated with bold arrows. Important phase I and II enzymes are listed with primary enzymes in bold.

Despite tamoxifen’s proven efficacy in reducing breast cancer recurrence and mortality [6], treatment outcomes vary considerably between individuals. One contributor to this variability is genetic variation in the enzymes involved in tamoxifen metabolism [7]. Tamoxifen is primarily metabolized by cytochrome P450 (CYP) enzymes, particularly CYP2D6 and CYP3A4/5, into its active metabolite, endoxifen, which exhibits most of its pharmacological activity (Figure 1) [8]. Endoxifen is subsequently inactivated by phase II enzymes, such as sulfotransferases (SULTs) and UDP-glucuronosyltransferases (UGTs) [8]. Genetic variants in these enzymes, especially *CYP2D6*, have been extensively studied for their impact on treatment outcomes. Despite numerous investigations, the contribution of pharmacogenetics to tamoxifen response remains uncertain due to inconsistent findings across cohorts, leaving a significant portion of the interindividual variability in treatment response unexplained [7]. Genetic variation in other phase I and phase II enzymes may also influence tamoxifen response, yet there is a dearth of research in this area.

Moreover, while most pharmacogenetic studies to date have focused on populations of European or Asian ancestry, individuals of African descent remain underrepresented [9]. The few studies that include individuals of African descent often focus on African American populations, who have different levels of admixture [10]. Given the high genetic diversity across African populations and the unique allele frequencies of pharmacogenes in these groups, the impact of pharmacogenetic variation on tamoxifen response remains largely unknown in native African populations.

Up to 40% of ER+ breast cancer patients may experience disease recurrence or death despite tamoxifen treatment [11]. Identifying genetic factors that influence tamoxifen efficacy in African populations is therefore critical to improving outcomes. Advancing this area of research is essential for enabling personalized treatment strategies, such as identifying non-responders, guiding dose adjustments, or selecting alternative therapies. These strategies have the potential to reduce breast cancer mortality across the continent.

This study contributes African data on tamoxifen pharmacogenetics through investigating the impact of genetic variation in tamoxifen-related pharmacogenes on clinical outcomes among South African breast cancer patients treated with tamoxifen.

## 2. Materials and Methods

### 2.1. Study Design, Setting, and Participants

This retrospective cohort study investigated the association between pharmacogenetic variation and disease-free survival (DFS) among breast cancer patients. The patient cohort and recruitment procedures have been described previously [12,13]. Briefly, between 2013 and 2018, women attending routine follow-up appointments at Groote Schuur breast clinic in Cape Town, South Africa, were recruited as part of a broader study investigating genetic contributors to shoulder pain and disability among breast cancer patients. The parent study was approved by the University of Cape Town Human Research Ethics Committee (UCT-HREC Ref: 312/2012).

From this cohort, DNA samples from 268 participants were available. In 2021, their hospital records were reviewed to extract clinical information and tamoxifen treatment outcomes. Eligibility criteria for the present analysis included a diagnosis of primary breast carcinoma, self-identification as Mixed or African Ancestry, and continuous tamoxifen treatment (20 mg/day) for at least four months, the estimated time to reach steady-state serum concentrations [14]. After applying the above criteria, 166 participants were included in the final analysis. No formal a priori sample size calculation was performed, and the sample size was determined by the availability of eligible participants and stored samples from the parent cohort. Ethical approval for this study was granted by the UCT-HREC (Ref: 795/2020), and all participants provided written informed consent.

### 2.2. Study Endpoint

The study endpoint was DFS, defined as the time from initiation of tamoxifen treatment to the first documented occurrence of disease progression within a 54-month follow-up period. Progression events included: local, regional, or distant breast cancer recurrence; new contralateral ductal carcinoma in situ or breast lesion; second primary invasive cancer; or death from any cause. Participants were followed from the initiation of tamoxifen therapy to the first occurrence of disease progression, loss to follow-up, or the end of a 54-month follow-up period. A 54-month follow-up period was used instead of the conventional 60 months (5 years) to align with institutional policy, which routinely discharges patients between months 54 and 60.

### 2.3. Gene Selection and Genetic Characterization

Eight pharmacogenes involved in the tamoxifen metabolism pathway were selected based on evidence from the Pharmacogenomics Knowledge Base (www.pharmgkb.org). Single nucleotide polymorphisms (SNPs) with a minor allele frequency (MAF) > 0.05 in the African (AFR) population were selected from the Ensembl database https://www.ensembl.org (accessed on 9 September 2020), supplemented by a targeted literature search. This exploratory approach prioritized African-enriched variants underrepresented in prior pharmacogenetic studies.

Detailed laboratory procedures for DNA extraction and genotyping have been described elsewhere [12,13]. Genotyping was performed using a custom SNP panel optimized by Inqaba Biotec (Pretoria, South Africa) for analysis on the MassARRAY^®^ System (Agena Bioscience, San Diego, CA, USA). Selected *SULT1A1* SNPs were genotyped using Sanger sequencing.

*CYP2D6* and *SULT1A1* copy number variations (CNVs) were also assessed to complement SNP-based genetic characterization. *CYP2D6* CNV was determined using the VeriDose^®^ *CYP2D6* CNV Panel (Agena Bioscience). *SULT1A1* copy number was quantified using a TaqMan^®^ Copy Number Assay (Hs04461762_cn, Applied Biosystems, Carlsbad, CA, USA), with RNase P as the two-copy reference gene. Copy number calls were generated using CopyCaller™ Software v2.1 (Applied Biosystems) via the comparative Ct method.

### 2.4. Statistical Analysis

Demographic and clinical characteristics were evaluated using STATA^®^ (Stata Corp, College Station, TX, USA) version 15.0 SE. Categorical variables are presented as counts and percentages (*n*, %), while continuous variables are reported as mean ± standard deviation (SD) or medians with interquartile ranges (IQRs), depending on data distribution. Normality of continuous variables was assessed using the Shapiro–Wilk test. Allele frequencies and Hardy–Weinberg equilibrium (HWE) were examined using SHEsis http://shesisplus.bio-x.cn/SHEsis.html (accessed on 12 May 2025), and linkage disequilibrium (LD) between SNPs was assessed using HaploView (Broad Institute, Cambridge, MA, USA) version 4.2.

Association analyses were conducted in R (R Foundation for Statistical Computing, Vienna, Austria) version 4.2.2 under an additive genetic model, with genotypes coded as 0, 1, or 2 according to the number of minor alleles. *CYP2D6* genotypes were translated into predicted metabolizer phenotypes using activity scores in accordance with Clinical Pharmacogenetics Implementation Consortium guidelines [15]. Since genotype phasing was not performed, star allele combinations for *CYP* genes were inferred from observed variant genotypes according to PharmVar https://www.pharmvar.org/ (accessed on 27 November 2024) definitions. Analysis was restricted to SNPs with an MAF > 0.05 in the study cohort.

Associations with DFS were assessed using Cox proportional hazards models. Participants who were lost to follow-up were censored at the time of last contact, while those who switched to alternative endocrine therapy after at least four months of continuous tamoxifen were not censored. All univariate models were adjusted for ethnicity, and variables with *p* ≤ 0.10 were carried forward into the multivariate model. Missing genotype data were rare and coded as “3”; these individuals were included in analyses but excluded from interpretation. For categorical clinical variables with missing values, an “unknown” category was created and included in regression analyses to preserve sample size, although results for this subgroup were not reported as they lacked clinical relevance. *p*-value < 0.05 was considered statistically significant. To account for multiple testing, Bonferroni correction was applied, yielding a corrected significance threshold of *p* < 0.002. Post hoc power calculations were conducted to evaluate the study’s ability to detect genetic associations of varying magnitudes.

## 3. Results

### 3.1. Participant Characteristics

Demographic and clinical characteristics of study participants are summarized in Table 1. A total of 166 participants were included in this study, of whom 139 (83.7%) were of Mixed Ancestry and 27 (16.3%) were of African Ancestry. Most participants (74.1%) had at least one comorbidity, with hypertension (49.4%) and diabetes (24.1%) being the most common. The highest number of comorbidities reported in a single patient was five (ischemic heart disease, gastro-esophageal reflux disease, hypertension, hypercholesterolemia, and osteoarthritis). The five most prescribed therapeutic drugs for treating comorbidities were acetaminophen (72.9%), tramadol (49.4%), hydrochlorothiazide (28.3%), ibuprofen (25.3%), and senna (25.3%). An overview of drugs known to inhibit CYP2D6 that were prescribed to patients in the study cohort is provided in Table S1.

The mean age at breast cancer diagnosis was 51.8 years (SD±10.3), with a median duration of tamoxifen use of 58 months (IQR, 41–61). A third of participants (*n* = 55) switched to alternative endocrine therapy, most commonly due to tamoxifen ineffectiveness (i.e., either disease progression or clinician concern for progression; 19.9%) or due to side effects (11.4%). Reasons for switching were undocumented in three patients. During the 54-month follow-up period, 26 women (15.7%) experienced disease progression.

The frequency distribution of genetic variants is summarized in Table S2. Four SNPs were monomorphic, and a further 14 had an MAF < 0.05 (Table S3); these SNPs were therefore excluded from subsequent analyses. As a result, 30 SNPs, the CYP2D6 metabolizer phenotype (derived from *CYP2D6* variants) and *SULT1A1* CNV were included in the analyses. Notably, *SULT1A1* exhibited unusually high copy numbers, with one participant of African Ancestry carrying seven copies and two participants (one each from the African and Mixed Ancestry groups) carrying eight copies.

### 3.2. Association of Demographic, Clinical, and Genetic Factors with DFS

Demographic, clinical, and genetic variables were evaluated for association with DFS using Cox proportional regression models adjusted for ethnicity (Table 2).

Among clinical factors, univariate analyses indicated that lymph node positivity and HER2 overexpression were significantly associated with reduced DFS. Receipt of chemotherapy and radiotherapy showed non-significant trends toward reduced DFS, whereas the presence of comorbidities demonstrated a trend toward improved DFS. In the multivariate model, only HER2 overexpression remained significantly associated with DFS (hazard ratio [HR] = 4.05; 95% confidence interval [CI] = 1.00–16.43; *p* = 0.050), although the wide CI indicates limited precision and the association should be interpreted cautiously. Of note, prescription of CYP2D6 inhibitors was not associated with DFS (HR = 0.45; 95% CI = 0.13–1.49; *p* = 0.190) and was therefore not included in the multivariate model.

With respect to genetic factors, *CYP2B6* rs3745274 and rs2279343 were grouped according to PharmVar allele definitions https://www.pharmvar.org/gene/CYP2B6 (accessed on 8 August 2025), which define the *9, *4 and *6 alleles. Due to unphased genotype data, it was not possible to distinguish between the *6/*6 and *6/*9, or the *1/*6 and *4/*9 diplotypes. Participants carrying two reduced-function alleles (*6/*6 or *6/*9) exhibited a trend toward reduced DFS in univariate analysis (HR = 2.30; 95% CI = 0.92–5.76; *p* = 0.076). In contrast, individuals with one reduced-function allele (*1/*6 or *4/*9) showed a nominal association with improved DFS compared with those carrying two functional alleles (*1/*1) in the multivariate model (HR = 0.35; 95% CI = 0.12–0.99; *p* = 0.049). No associations were observed between DFS and CYP2D6-predicted phenotype (Table S4).

The *UGT1A4* rs11888492 SNP exhibited a protective effect on DFS, with heterozygous individuals showing improved DFS compared with those homozygous for the reference allele. Although significant in univariate analysis, this association persisted only as a non-significant trend after multivariable adjustment (HR = 0.43; 95% CI = 0.16–1.15; *p* = 0.093). In contrast, several phase II enzyme variants, namely *SULT1A1* (rs4149393, rs4149394, and rs1042157), *SULT1E1* rs3775779, and *SULT2A1* rs11569679, were associated with poorer outcomes. Due to strong LD among the three *SULT1A1* SNPs (D′ = 1; R^2^ = 0.9), rs4149393 was selected as a representative marker for subsequent analyses. *SULT1A1* heterozygotes experienced significantly reduced DFS in univariate analysis, although this association persisted only as a non-significant trend in the multivariate model (HR = 2.52; 95% CI = 0.90–7.07; *p* = 0.080). No disease progression events were observed among participants homozygous for the *SULT1A1* variant alleles, precluding HR estimation for these genotypes. These three *SULT1A1* variants deviated from HWE in the Mixed Ancestry group (Table S5), potentially reflecting CNV-related effects. Given their biological relevance and the exploratory nature of this study, these variants were retained in analyses.

Heterozygotes for *SULT1E1* rs3775779 demonstrated reduced DFS in univariate analysis, which remained nominally significant in the multivariate model (HR = 2.52; 95% CI = 1.02–6.18; *p* = 0.044). Of note, none of the 18 carriers of the *SULT2A1* rs11569679A allele experienced disease progression during follow-up. This SNP could not be included in analyses due to the inability to estimate HRs.

Post hoc power calculations indicated that, assuming an MAF ≥ 20%, the study had greater than 70% power to detect HRs of 2.5 or higher, as observed for *SULT1E1* (rs3775779) and *CYP2B6* (rs3745274 and rs2279343). After Bonferroni correction for multiple testing, none of the evaluated variables remained statistically significant.

## 4. Discussion

Breast cancer survival disparities across the world are driven by variability in the stage at which the disease is diagnosed or patient presentation at a healthcare facility [16]. Tamoxifen remains a mainstay of endocrine therapy in many African settings [4], yet its clinical benefits are highly variable. While pharmacogenetic influences on tamoxifen response have been extensively investigated in European and Asian populations with conflicting results, the question of their role among African populations remains largely unanswered [9]. This study contributes African data by evaluating genetic variation in phase I and phase II metabolizing enzymes in a South African cohort of African and Mixed Ancestry women.

Among the clinical variables assessed, HER2 overexpression remained significantly associated with reduced DFS in multivariate analysis, consistent with a prior study [17], although the wide CI indicates limited precision. Prior reports in African women have shown significant associations between *CYP2D6* genotypes/phenotypes and plasma endoxifen levels [18], supporting a plausible role for *CYP2D6* in tamoxifen response. However, in this South African cohort, CYP2D6-predicted phenotype was not significantly associated with DFS. Although one study among Egyptians reported a significant association between *CYP2D6* and tamoxifen response [19], our findings align with multiple studies in other populations that have failed to detect a consistent link between *CYP2D6* variation and clinical outcomes [20].

Our analysis suggests a possible association between *CYP2B6* genotype and DFS, with carriers of one reduced-function allele (*1/*6 or *4/*9) showing a nominal association with improved DFS. This finding is unexpected given CYP2B6’s relatively minor role in producing 4-hydroxytamoxifen and contrasts with prior studies in European cohorts that reported no significant associations with clinical outcomes [21,22,23]. The apparent protective effect in *CYP2B6* heterozygotes may reflect confounding, or a heterozygote advantage. Alternatively, it is possible that co-medications, which can induce or inhibit *CYP2B6* expression, modulate enzyme activity such that heterozygous individuals may functionally resemble normal or poor expressors depending on their co-medication profile. This possibility underscores the need for caution in interpretation, and these associations require replication in larger, prospective cohorts before firm conclusions can be drawn.

Tamoxifen and its metabolites are primarily inactivated via glucuronidation, with UGT1A4 playing a major role in this process [8]. In the present study, the *UGT1A4* rs11888492 intronic variant showed a trend toward improved DFS in the multivariate analysis. In silico prediction (https://hsf.genomnis.com/home) suggested potential effects on splicing, which may reduce enzyme activity and, in turn, lead to increased circulating endoxifen levels. However, this finding was not statistically significant after adjustment and remains exploratory.

Three *SULT1A1* variants (rs4149393, rs4149394, and rs1042157) showed a trend toward reduced DFS in the multivariate analysis. This observation is biologically intriguing, as reduced SULT1A1 activity resulting from these variants would be expected to prolong endoxifen exposure and potentially improve outcomes [24]. However, similar paradoxical associations have been reported for the SULT1A1*2 variant, which also decreases SULT1A1 activity [25,26,27]. Several biological mechanisms have been proposed to explain these unexpected observations [25]. Other possible explanations include effects on tumor biology [28], altered estrogen sulfation [29], or LD with other functional loci.

The lack of HWE for these variants in the Mixed Ancestry group is likely attributable to *SULT1A1* CNV, as standard HWE calculations assume diploid genotype structure. Similar deviations have been reported in other studies of *SULT1A1* [30,31,32]. High copy numbers are common in Africans [33], and gene copies ranging from zero to six have been reported before [34]. In addition, the highly admixed nature of the South African Mixed Ancestry population may contribute to deviations from HWE due to population substructure. To our knowledge, we are the first to report *SULT1A1* copy numbers of seven and eight. Although no association was observed between *SULT1A1* copy number and DFS in our cohort, the functional impact of very high copy numbers on tamoxifen response remains a subject for future studies, particularly in African populations.

The *SULT1E1* rs3775779 intronic variant, predicted to activate a cryptic acceptor site, was associated with reduced DFS in multivariate analysis. Given that SULT1E1 has the highest affinity for estrogens within the SULT family and is crucial for regulating estrogen bioavailability [29], variation in this gene may affect tamoxifen efficacy through altered estrogen metabolism. Reduced SULT1E1 activity may impair estrogen clearance, increasing the availability of active estrogens that can fuel ER+ tumor growth, thereby diminishing tamoxifen’s anti-estrogenic effect. To date, *SULT1E1* has not been studied in tamoxifen pharmacogenetics, and our results support its inclusion in future large-scale studies, particularly in African ancestry populations where allele frequencies and LD structures may differ substantially from other groups.

For *SULT2A1*, none of the 18 carriers of rs11569679 experienced disease progression, suggesting a potential protective effect. This variant disrupts the enzyme’s dimerization motif [35], which may reduce enzymatic activity and lead to prolonged endoxifen exposure. However, the small sample size limits the reliability of this observation, and it should be interpreted with caution. Notably, rs11569679 is rare globally but enriched in populations of African descent, highlighting the importance of targeted functional studies and replication in larger African cohorts.

Our study is not free from limitations. This study may be subject to selection bias, as only participants who underwent breast surgery were included (as part of the broader study), excluding those treated with tamoxifen alone, without surgery. This may limit generalizability to all tamoxifen users. Due to the retrospective nature of this study, adherence information could not be accurately captured, as adherence was not routinely documented beyond occasional physician notes based on patient self-report. While powered to detect large effects for common variants, the study was underpowered for rare alleles, and some genotype groups had very low carrier numbers, limiting statistical precision. In addition, the relatively small number of disease progression events (*n* = 26) may have contributed to wide CIs and reduced the reliability of HR estimates. Therefore, the findings should be interpreted as exploratory and require validation in larger cohorts. Functional interpretations for certain variants relied on in silico predictions and literature from non-African populations, which may not fully capture their effects in this context. Although prescription of CYP2D6 inhibitors was considered, the potential influence of other concomitant medications on treatment outcomes cannot be excluded.

Given the distinct allele frequencies, LD structures, and CNV patterns observed in Africans, a “one-size-fits-all” pharmacogenetic model derived from European or Asian datasets is unlikely to be applicable to African populations. Larger, prospective African cohort studies are needed to validate the findings from our study. Importantly, future work should incorporate pharmacokinetic measurements of tamoxifen and its metabolites, and capture adherence data. Expanding to genome-wide or exome-wide analyses could reveal additional variants, including those outside classical metabolic pathways. Functional validation, particularly for African-specific or African-enriched alleles, will be key to understanding their biological and clinical relevance.

## 5. Conclusions

This exploratory study highlights the potential contribution of pharmacogenetic variation beyond *CYP2D6* to tamoxifen response in African populations, underscoring the need for inclusive research that reflects Africa’s genetic diversity. While none of the observed associations remained significant after correction for multiple testing, the findings raise the possibility that phase II enzymes play a larger role in tamoxifen pharmacogenetics among African populations than previously appreciated. These results support the expansion of African-led tamoxifen pharmacogenetic studies to incorporate a broader range of metabolic genes and variants enriched in African populations, with the goal of improving precision endocrine therapy for African breast cancer patients.

## Figures and Tables

**Table 1:** Demographic and clinical characteristics of South African breast cancer patients on tamoxifen treatment.

Characteristic	Value
Ethnicity, *n* (%)	
Mixed	139 (83.7)
African	27 (16.3)
BMI, median (IQR)	29.5 (26.6–33.7)
Smoking history, *n* (%)	
Yes	65 (39.2)
No	87 (52.4)
Unknown	14 (8.4)
Alcohol consumption, *n* (%)	
Yes	29 (17.5)
No	106 (63.8)
Unknown	31 (18.7)
Co-morbidities, *n* (%)	
Yes	123 (74.1)
No	37 (22.3)
Unknown	6 (3.6)
Top co-morbidities, *n* (%)	
Hypertension	82 (49.4)
Diabetes	40 (24.1)
Asthma	17 (10.2)
Hypercholesterolemia	13 (7.8)
Menopausal status ^a^, *n* (%)	
Yes	66 (39.7)
No	69 (41.6)
Unknown	31 (18.7)
Family history of cancer, *n* (%)	
Yes	78 (47.0)
No	78 (47.0)
Unknown	10 (6.0)
Age at diagnosis (years), mean (±SD; range)	51.8 (10.3; 30.0–74.0)
Surgery, *n* (%)	
Mastectomy	134 (80.7)
WLE	32 (19.3)
Chemotherapy, *n* (%)	
Yes	128 (77.1)
No	38 (22.9)
Radiotherapy, *n* (%)	
Yes	100 (60.2)
No	66 (39.8)
Histological classification, *n* (%)	
IDC	148 (89.2)
ILC	9 (5.4)
Both	6 (3.6)
Unknown	3 (1.8)
Tumor grade ≥ G2, *n* (%)	
Yes	118 (71.1)
No	38 (22.9)
Unknown	10 (6.0)
Tumor stage ≥ T2, *n* (%)	
Yes	116 (69.9)
No	47 (28.3)
Unknown	3 (1.8)
Lymph node-positive, *n* (%)	
Yes	91 (54.8)
No	75 (45.2)
Estrogen receptor-positive ^b^, *n* (%)	
Yes	155 (93.4)
No	11 (6.6)
Progesterone receptor-positive, *n* (%)	
Yes	19 (11.5)
No	6 (3.6)
Unknown	141 (84.9)
HER2-positive ^c^, *n* (%)	
Yes	53 (31.9)
No	52 (31.3)
Unknown	61 (36.8)
Tamoxifen duration (months), median (IQR)	58 (41.0–61.0)
Reason for alternative endocrine therapy, *n* (%)	
Ineffective ^d^	33 (19.9)
Side effects	19 (11.4)
Unknown	3 (1.8)
No switch	111 (66.9)
Disease progression ^e^, *n* (%)	
Yes	26 (15.7)
No	140 (84.3)

Notes: ^a^ Perimenopausal women were included in the premenopausal classification. ^b^ Determined using the Allred score, where a score of 3–8 is deemed positive, and a score of 0–2 is deemed negative. ^c^ Based on immunohistochemistry results. ^d^ Ineffective tamoxifen treatment refers to disease progression or fear of disease progression, which resulted in a switch from tamoxifen to an alternative endocrine therapy. ^e^ Disease progression refers to individuals whose disease progressed within four and a half years of initiating tamoxifen (including individuals who switched to alternative therapies during this period). Disease progression was defined as the occurrence of any of the following events: a breast cancer recurrence (local, regional, or distant), development of a new contralateral DCIS or breast lesion, diagnosis of a second primary invasive cancer, or death from any cause. Abbreviations: BMI, body mass index; G2, tumor grade II; HER2, human epidermal growth factor receptor 2; IDC, invasive ductal carcinoma; ILC, invasive lobular carcinoma; IQR, interquartile range; SD, standard deviation; T2, tumor stage II; WLE, wide local excision

**Table 2:** Univariate and multivariate Cox regression of key demographic, clinical, and genetic factors associated with disease-free survival, adjusted for ethnicity.

	No. of Patients N = 166	No. of Progression Events N = 26	Univariate Model			Multivariate Model		
			HR	95% CI	*p*-Value	HR	95% CI	*p*-Value
Lymph node-positive								
No	75	6	1.00			1.00		
Yes	91	20	2.93	1.17–7.30	**0.021**	2.47	0.90–6.79	0.079
HER2-positive								
No	52	4	1.00			1.00		
Yes	53	13	3.41	1.10–10.52	**0.033**	4.05	1.00–16.43	**0.050**
Chemotherapy								
No	38	2	1.00			1.00		
Yes	128	24	3.51	0.82–15.02	0.090	1.53	0.29–8.06	0.616
Radiotherapy								
No	66	6	1.00			1.00		
Yes	100	20	2.40	0.96–5.97	0.061	2.31	0.81–6.62	0.119
Co-morbidities								
No	37	10	1.00			1.00		
Yes	123	16	0.46	0.21–1.04	0.062	0.62	0.25–1.53	0.304
*CYP2B6* ^a^								
*1/*1	77	13	1.00			1.00		
*1/*6 or *4/*9	69	6	0.48	0.18–1.27	0.138	0.35	0.12–0.99	**0.049**
*6/*6 or *6/*9	20	7	2.30	0.92–5.76	0.076	1.84	0.65–5.20	0.250
*UGT1A4* rs11888492								
C/C	91	17	1.00			1.00		
C/G	70	7	0.34	0.14–0.86	**0.023**	0.43	0.16–1.15	0.093
G/G	15	2	0.33	0.07–1.63	0.175	0.29	0.06–1.39	0.121
*SULT1A1* rs4149393 ^b^								
A/A	74	7	1.00			1.00		
A/G	87	19	2.48	1.04–5.91	**0.041**	2.52	0.90–7.07	0.080
G/G	5	-	-	-	-	-	-	-
*SULT1E1* rs3775779								
A/A	109	14	1.00			1.00		
A/T	50	11	2.07	0.92–4.68	0.079	2.52	1.02–6.18	**0.044**
T/T	7	1	1.46	0.19–11.38	0.721	6.19	0.52–73.70	0.149

Notes: Only variables with *p*-values ≤ 0.10 in univariate analyses are shown; these variables were included in the multivariable model. *p*-values ≤ 0.05 are indicated in bold. ^a^ *CYP2B6*: groups were assigned according to PharmVar allele definitions. rs3745274 corresponds to *9 and rs2279343 to *4. When both variants co-occur, they define the *6 haplotype. Due to unphased genotype data, it was not possible to distinguish *1/*6 from *4/*9. ^b^ *SULT1A1*: rs4149393 was included in the model as a representative marker for rs4149393, rs4149394, and rs1042157 due to strong linkage disequilibrium among the variants (D′ = 1; R^2^ = 0.9). Abbreviations: CI, confidence interval; HER2, human epidermal growth factor receptor 2; HR, hazard ratio.

## Data Availability

The data that support the findings of this study are available from the corresponding author [C.D.] upon reasonable request.

## Supplementary Materials

The following supporting information can be downloaded at: https://www.mdpi.com/article/10.3390/jpm16040188/s1, Table S1: CYP2D6 inhibitors prescribed to South African breast cancer patients on tamoxifen treatment; Table S2: Frequency distribution of Cytochrome P450 (*CYP*), UDP-glucuronosyltransferase (*UGT*) and sulfotransferase (*SULT*) alleles in South African breast cancer patients and other world populations; Table S3: Pharmacogenetic variants with frequency < 0.05 in the study population and excluded from association analyses; Table S4: Univariate analysis of CYP2D6-predicted phenotype in relation to disease-free survival, adjusted for ethnicity; Table S5: HardyWeinberg equilibrium *p*-values for study variants.
