# Supplementary material for: Genetic Variation in CYP2B6, UGT1A4 and Sulfotransferases Is Associated with Disease-Free Survival in South African Breast Cancer Patients Treated with Tamoxifen
Source: J Pers Med. 2026 Mar 31;16(4):188. doi: 10.3390/jpm16040188 (PMC13117320; doi:10.3390/jpm16040188)
Supplement: Supplementary file 1 [file jpm-16-00188-s001.zip › jpm-4152740-supplementary.pdf]

Table S1. CYP2D6 inhibitors prescribed to South African breast cancer patients on tamoxifen treatment.

CYP2D6 inhibitor	Patients, n (%) N = 166
Metoclopramide	20 (12.0)
Diphenhydramine	9 (5.4)
Citalopram	7 (4.2)
Promethazine	6 (3.6)
Cimetidine	4 (2.4)
Fluoxetine	3 (1.8)
Chlorphenamine	1 (0.6)
Terbinafine	1 (0.6)
Venlafaxine	1 (0.6)
Total *	52 (31.3)

Notes: * The total does not reflect individual patients, as individuals may have been prescribed different inhibitors at different times.

Table S2. Frequency distribution of Cytochrome P450 (*CYP*), UDP-glucuronosyltransferase (*UGT*) and sulfotransferase (*SULT*) alleles in South African breast cancer patients and other world populations.

Gene	dbSNP HGVS name	Variant ID	Position and alleles	Location in gene	Allele	This study		gnomAD*	
						Mixed Ancestry N = 278	African Ancestry N = 54	African or African American	European
CYP2D6	NM_000106.6	rs769258	31G>A	Exon	A	0.018	0	0.008	0.055
		rs28371696	77G>A	Exon	A	0.011	0	0.019	<0.001
		rs1065852	100C>T	Exon	T	0.233	0.092	0.124	0.223
		rs28371703	271C>A	Exon	A	0.032	0	0.038	0.192
		rs28371706	320C>T	Exon	T	0.058	0.314	0.181	0.001
		rs61736512	406G>A	Exon	A	0.032	0.074	0.091	0.001
		rs28371710	463G>A	Exon	A	0.021	0.074	0.044	<0.001
		rs3892097	506-1G>A	Intron	A	0.098	0	0.077	0.199
		rs16947	886C>T	Exon	T	0.441	0.537	0.518	0.335
		rs28371725	985+39G>A	Intron	A	0.083	0	0.026	0.096
		rs59421388	1012G>A	Exon	A	0.032	0.074	0.086	0.001
		rs1058172	1094G>A	Exon	A	0.014	0	0.014	0.092
CYP3A4	NM_017460.6	rs2740574	-392G>A	Promoter	G	0.266	0.740	0.641	0.035
		rs35599367	522-191C>T	Intron	T	0.017	0	0.009	0.049
		rs2242480	1026+12G>A	Intron	A	0.496	0.851	0.733	0.094
CYP3A5	NM_000777.5	rs776746	219-237A>G	Intron	A	0.435	0.870	0.695	0.069
		rs10264272	624G>A	Exon	A	0.075	0.314	0.124	0.001
		rs41303343	1035dup (A>AA)	Exon	AA	0.025	0.074	0.101	<0.001
		rs15524	*14T>C	3′ UTR	C	0.352	0.722	0.585	0.076
CYP2B6	NM_000767.5	rs8192709	64C>T	Exon	T	0.046	0.074	0.036	0.054
		rs3745274	516G>T	Exon	T	0.330	0.314	0.370	0.237
		rs2279343	785A>G	Exon	G	0.338	0.296	0.356	0.252
		rs28399499	983T>C	Exon	C	0.025	0.074	0.070	<0.001
		rs3211371	1459C>T	Exon	T	0.071	0	0.026	0.122
UGT1A4	NM_007120.3	rs869283	-1180G>A	Promoter	G	0.489	0.092	0.285	0.621

		rs3732218	-163G>A	Promoter	A	0.140	0.018	0.104	0.088
		rs3892221	31C>T	Exon	T	0.017	0.111	0.043	<0.001
		rs2011425	142T>G	Exon	G	0.133	0.018	0.100	0.087
		rs45540231	526A>T	Exon	T	0.017	0.074	0.063	<0.001
		rs4148328	1307+574C>T	Intron	T	0.341	0.092	0.177	0.381
		rs11888492	1308-934C>G	Intron	G	0.248	0.574	0.372	0.105
		rs8330	*440G>C	3′ UTR	G	0.291	0.314	0.412	0.230
SULT1A1	NM_001055.4	rs4149393	595-38T>C	Intron	C	0.305	0.222	0.235	0.391
		rs4149394	595-17G>A	Intron	A	0.305	0.222	0.234	0.390
		rs1042028	638G>A	Exon	A	0.251	0.240	0.236	0.337
		rs1801030	667G>A	Exon	G	0.075	0.222	0.266	0.001
		rs28374453	739T>C	Exon	C	0.046	0.111	0.067	0.002
		rs6839	*14A>G	3′ UTR	G	0.266	0.203	0.224	0.352
		rs1042157	*85C>T	3′ UTR	T	0.302	0.222	0.237	0.392
SULT1E1	NM_005420.3	rs3736599	-64G>A	5′ UTR	A	0.187	0.259	0.194	0.096
		rs3822172	-9-469G>A	Intron	G	0.289	0.481	0.358	0.146
		rs3775775	369+1653T>C	Intron	C	0.136	0.314	0.222	0.094
		rs1220702	497-10C>G	Intron	G	0.071	0.111	0.095	0.126
		rs3775779	772+672A>T	Intron	A	0.215	0.074	0.141	0.314
SULT2A1	NM_003167.4	rs11569681	187G>C	Exon	C	0.010	0	0.063	<0.001
		rs4149448	345+477T>C	Intron	C	0.154	0.259	0.185	0.112
		rs2547231	472+284G>T	Intron	G	0.082	0.092	0.095	0.163
		rs11569679	781G>A	Exon	A	0.057	0.055	0.120	<0.001
		rs11569678	*77A>T	3′ UTR	T	0.043	0.055	0.051	<0.001

Notes: [*]Data from gnomAD genomes v4.1 published on Ensembl (https://www.ensemb.org/index.html). Monomorphic SNPs (rs55785340, rs4987161, rs28365083, rs33980385) are not shown. **Abbreviations:** 3′ UTR, three prime untranslated region; 5′ UTR, five prime untranslated region; dbSNP, The Single Nucleotide Polymorphism Database; gnomAD, The Genome Aggregation Database; HGVS, Human Genome Variation Society.

Table S3. Pharmacogenetic variants with frequency < 0.05 in the study population and excluded from association analyses.

Gene	Variant ID		
CYP2D6	rs769258<br>rs61736512<br>rs1058172	rs28371696<br>rs28371710	rs28371703<br>rs59421388
CYP3A4	rs35599367	**rs55785340**	**rs4987161**
CYP3A5	rs41303343	**rs28365083**	
CYP2B6	rs28399499	**rs33980385**	
UGT1A4	rs3892221	rs45540231	
SULT2A1	rs11569681	rs11569678	

Notes: Variants in bold are monomorphic.

Table S4. Univariate analysis of CYP2D6-predicted phenotype in relation to disease-free survival, adjusted for ethnicity.

	No. of patients	HR	95% CI	P-value
EM	88	1.00		
IM	52	0.72	0.29-1.77	0.472
UM	2	3.62	0.47-27.86	0.216
PM	7	0.98	0.13-7.50	0.982
UM + EM + IM	142	1.00		
PM	7	1.04	0.14-7.79	0.973

Abbreviations: CI, confidence interval; EM, extensive metabolizer; HR, hazard ratio; IM, intermediate metabolizer; PM, poor metabolizer; UM, ultrarapid metabolizer.

Table S5. Hardy–Weinberg equilibrium *p*-values for study variants.

Gene	Variant ID	Mixed Ancestry	African Ancestry
CYP3A4	rs2740574	0.350	0.853
	rs2242480	0.671	0.534
CYP3A5	rs776746	0.565	0.351
	rs10264272	0.335	**0.038**
	rs15524	0.311	0.299
CYP2B6	rs8192709	0.563	0.678
	rs3745274	0.287	0.546
	rs2279343	0.424	0.206
	rs3211371	0.722	-
UGT1A4	rs869283	0.090	0.078
	rs3732218	0.374	0.922
	rs2011425	0.693	0.922
	rs4148328	0.930	0.596
	rs11888492	0.477	0.480
	rs8330	0.366	0.237
SULT1A1	rs4149393	**0.001**	0.138
	rs4149394	**0.001**	0.138
	rs1042028	**0.030**	0.099

	rs1801030	0.335	0.711
	rs28374453	0.563	0.516
	rs6839	0.095	0.184
	rs1042157	**0.002**	0.552
	rs3736599	0.233	0.414
	rs3822172	0.867	0.180
SULT1E1	rs3775775	0.313	0.772
	rs1220702	0.722	0.194
	rs3775779	0.792	0.678
	rs4149448	0.277	0.235
SULT2A1	rs2547231	0.288	0.596
	rs11569679	0.399	0.760

Notes: *P*-values ≤ 0.05 are indicated in bold.
